# Influence of Pressing Temperatures on Physical–Mechanical Properties of Wood Particleboards Made with Urea-Formaldehyde Adhesive Containing Al_2_O_3_ and CuO Nanoparticles

**DOI:** 10.3390/polym16121652

**Published:** 2024-06-11

**Authors:** Luana Cristal Lirya Silva, Felipe Oliveira Lima, Victor Almeida De Araujo, Herisson Ferreira Dos Santos, Francisco Antonio Rocco Lahr, André Luis Christoforo, Higor Rogério Favarim, Cristiane Inácio de Campos

**Affiliations:** 1 Science and Engineering Faculty, São Paulo State University, Guaratinguetá 12516-410, Brazil; 2 Science and Engineering Institute, São Paulo State University, Itapeva 18409-010, Brazil; 3 Exact Sciences & Technology Center, Federal University of São Carlos, São Carlos 13565-905, Brazil; 4 Campus of Ariquemes, Federal Institute of Education, Science and Technology of Rondônia, Ariquemes 76870-000, Brazil; 5 São Carlos School of Engineering, University of São Paulo, São Carlos 13566-590, Brazil

**Keywords:** wood-based panel, density, thermal property, physical property, mechanical property, pressing temperature, nanomaterials, polymeric adhesive

## Abstract

Particleboards have gained attention in the global market. Understanding their physical–mechanical behavior in the current technological context is essential due to adhesive polymerization, which depends on variables such as pressing time and temperature. Today, the use of nanoparticles has become a plausible option for improving the properties of polymers used in wood-based composites. This study evaluates the influences of the addition of non-commercial 0.5% aluminum oxide (Al_2_O_3_) and aluminum oxide copper (CuO) nanoparticles using a greener route with a lower environmental impact obtaining a urea-formaldehyde (UF)-based polymeric adhesive to manufacture particle composites of *Eucalyptus urophylla* var. *grandis* wood. Regarding characterizations, the resin properties analyzed were viscosity, gel time, and pH, as well as panel properties, including density, moisture content, thickness swelling, modulus of elasticity, modulus of rupture, and thermal conductivity. The results were compared with scientific publications and standards. The addition of nanoparticles interfered with viscosity, and all treatments indicated a basic pH. It was not possible to determine the gel time after 10 min. Nanoparticles added to the polymers in the internal layer did not cause an improvement in the swelling properties in terms of thickness, with no significant statistical difference for density and moisture content. The increase from 150 °C to 180 °C may have caused an improvement in all physical–mechanical properties, indicating that the higher temperature positively influenced the polymerization of the formaldehyde-based adhesive. Therefore, the additions of both nanoparticles (0.5% in each condition) led to a limitation in the material influence with respect to physical–mechanical performance.

## 1. Introduction

Particleboards have been increasingly used, with a favorable trend for the coming decades [1,2]. Due to the increase in the market competitiveness for bio-based particulate composites, understanding their physical and mechanical behavior is paramount in the current technological context [3].

Particleboard (PB) is a composite manufactured with wood particles aggregated by synthetic adhesives through reconstitution and consolidation of the material using heating and pressure in a forming press. Urea-formaldehyde is one of the most usable resins in particleboard production [4].

Therefore, it is important to understand that the performance of a wood composite is directly related to the polymerization of the adhesive, which can depend on variables such as pressing time and temperature [3,5,6,7].

Thus, the composite properties can still be made worse by inefficient heat transfer, as the physical–mechanical performance is directly related to an efficient constituent interaction consolidated by heat transfer from the heated press along the panel thickness [6,8]. It is worth highlighting that pressing is one of the different manufacturing stages in panel production, because the reduction of costs involved in the production must be prioritized as a leading objective of every manufacturer.

The low thermal conductivity of wood is responsible for problems in heat transfer from pressed panels under the influence of temperature. In this sense, new technological trends verify the possibility of better performance with the use of nanometric-scale materials. Nanomaterials can present new behaviors and properties when compared to materials on a macroscopic scale [9]. Thus, the heat-conducting nature of nanoparticles produced with metals can be used to improve heat transfer to the innermost layer of the panel mat, which contributes to the polymerization of the resin and, consequently, impacts the properties of the composite [10,11,12]. Aluminum oxide nanoparticles have been used as reinforcement in polymer composites for multiple purposes [13]. Reinforcement with aluminum oxide nanoparticles in various thermosetting resins has been researched for the development of value-added products [14]. Researchers from different countries have been studying the application of nanoparticles in polymeric adhesives for the manufacture of wood composites. Some studies present the addition of nanomaterials to urea-formaldehyde resins, reporting improvements in mechanical properties, directly affecting the swelling of panels when in contact with water, and reducing the formaldehyde emission [15,16,17].

According to Cadermatori et al. [15], thermodynamic analysis revealed that Al_2_O_3_ is an effective additive for urea-formaldehyde (UF), maintaining crucial curing parameters such as the vitrification point and gel temperature. Additionally, Al_2_O_3_ nanoparticles basically reduced formaldehyde emissions during the UF resin curing process at elevated temperatures and achieved up to a 14% reduction in thickness at room temperature.

Zhang et al. [18] concluded that aluminum oxide nanoparticles, in percentages of 0% to 4%, when used together with phenol-formaldehyde adhesive in plywood pressed at 140 °C, showed an ability to accelerate and optimize the curing process, which increases manufacturing efficiency and reduces energy consumption in the production process.

Gupta et al. [19] studied a mixture of aluminum oxide with urea-formaldehyde resin, in percentages of 0.5%, 1%, and 1.5%, and they verified a reduction in curing time due to the greater thermal conductivity of the medium-density fiberboard matrix, as well as the significant improvement of the physical and mechanical properties with the addition of Al_2_O_3_ nanoparticles. Taghiyari and Bibalan [20] studied the use of Cu nanoparticles in particleboards with urea-formaldehyde adhesive, in proportions of 100 and 150 mL/kg based on the weight of dry particles, and better polymerization of the resin in the inner layer of the panel was verified due to improved heat transfer from copper nanoparticles and a very significant reduction in hot-pressing time.

Unlike other reported works that utilize commercial nanoparticles (Al_2_O_3_, CuO, and ZnO) and formaldehyde-based adhesives commonly utilized in panel production, our main goal in this study involves the use of nanoparticles from an alternative route for nanoparticle synthesis with lower costs and environmental impacts.

For this, this work analyzed two pressing temperature levels (150 °C to 180 °C) for wood particleboards and the effects on the curing of polymeric resin (UF) reinforced with (0.5%) and without aluminum oxide (Al_2_O_3_) and copper oxide (CuO) nanoparticles, verifying their physical–mechanical properties for a more environmentally friendly option.

## 2. Materials and Methods

### 2.1. Panel Configurations and Characterization of Nanoparticles

#### 2.1.1. Materials

The following materials related to panels and nanoparticles were included:

- Particles of Eucalyptus urophylla var. grandis;
- Urea-formaldehyde adhesive, with 68% solid content and pH 8 (Hexion, Curitiba, Brazil);
- Ammonium sulfate;
- Al_2_O_3_ nanoparticles (own laboratory production, Itapeva, Brazil);
- CuO nanoparticles (own laboratory production, Itapeva, Brazil);
- X-ray diffraction equipment (Bruker AXS D8, Billerica, MA, USA);
- Pneumatic press (Hidralmaq HMP 80 ton model, Araraquara, Brazil);
- K thermocouple (Hikari HKP01 model, São Paulo, Brazil);
- Gluing rotational machine (Marconi MA686, Piracicaba, Brazil);
- Device for data acquisition and computer.

#### 2.1.2. Methods

For panels, particles of *Eucalyptus urophylla* var. *grandis* were used as the main raw material, urea-formaldehyde adhesive with 65% solid content as the gluing agent, and ammonium sulfate as the catalyst with 20% solid content, as well as 0.5% aluminum oxide nanoparticles (Al_2_O_3_) and 0.5% copper oxide (CuO) nanoparticles. Water was utilized in the resin preparation, as well as in the water absorption test.

In the characterization of nanoparticles, the calcined material was analyzed by X-ray diffraction. Subsequently, microstructural analysis was carried out with a scanning electron microscope (SEM) to determine the shape of the synthesized material, as well as the size of the particles before the panel production. Scherrer’s analysis was carried out.

The panels were manufactured rigorously according to the methodology previously utilized by Silva et al. [8] and Lima [21]. All stages of the panel manufacturing process in the laboratory and the characterization procedures followed the recommendations given by the ABNT NBR 14810-3:2018 [22].

Regarding materials, wood, adhesive, and additives were obtained by confidential donations through Brazilian manufacturers. To produce each panel with a calculated nominal density of 720 kg/m^3^ considering nominal dimensions (40 cm × 40 cm × 1.3 cm), 1500 g of *Eucalyptus urophylla* var. *grandis* wood particles was used, which were properly classified according to 5, 9, 16, 35, and 60 mesh. Of these proportions, 5, 9, and 16 mesh were used for the single inner layer, while 35 and 60 mesh were used for the two outer layers.

Urea-formaldehyde (UF) adhesive with a solids content of 65% was prepared under a dosage based on the weight of the particles, using 10% for the external layers and 8% for the internal layer, where 0.5% nanoparticles were utilized in the inner layer of the panel. The nanoparticles were obtained in partnership with other researchers and were produced by the sol–gel protein method. A protein precursor was dissolved at 40 °C together with the metal salts in a stoichiometric proportion. The solution was dried in an oven until it formed a spongy structure, which was then burned and calcined to form the nanomaterial, as performed by Silva et al. [8].

**Figure 1:**
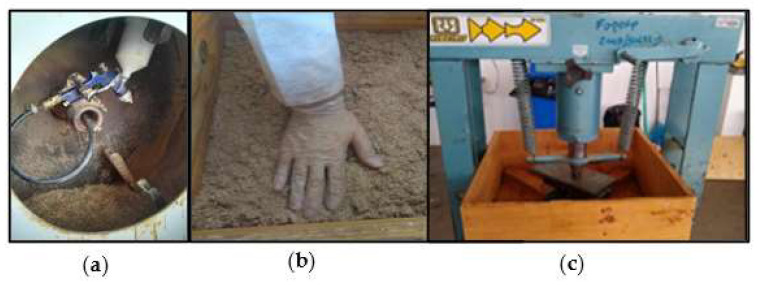
Panel production stages: (**a**) resin spraying, (**b**) formation of mat with wood particles, and (**c**) pre-pressing.

Wood particles were dried in a chamber (103 °C ± 2) to reach 3% moisture content and properly weighed in a proportion of 25–50–25% between layers. The resin was sprayed onto the outer- and inner-layer particles on a rotary gluing machine (Figure 1a).

The particle mat was assembled in a forming box (40 cm × 40 cm) (Figure 1b), and the formation was finalized through cold pre-pressing in a pneumatic press (Figure 1c) for 300 s at 0.3 MPa. After the pre-pressing stage, the analysis of temperature variation was performed by a type K thermocouple inserted into the innermost layer of the panel.

This thermocouple was coupled to a data acquisition device (DAQ) to obtain the temperature x time graph. 

**Figure 2:**
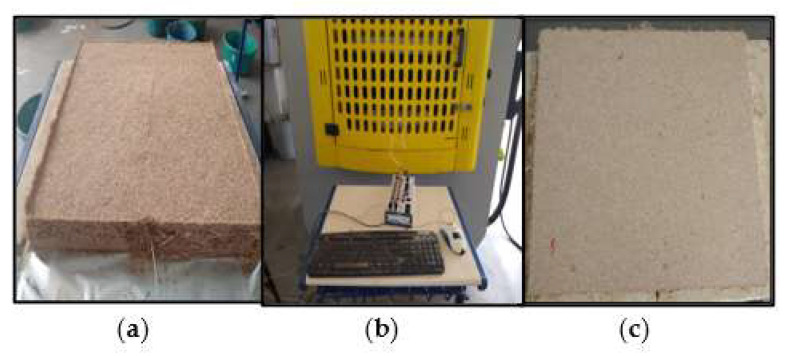
Panel production stages: (**a**) pre-pressed mat, (**b**) hot pressing, and (**c**) particleboard.

From pre-pressed mats (Figure 2a), the hot-pressing process was started with a total cycle of 600 s, with two pressure releases with a duration of 30 s each (Figure 2b). The pressure was set at 4 MPa with variations in temperature, at 150 °C and 180 °C. Thus, particleboards were successfully obtained, with an average thickness of 13 mm, under different manufacturing conditions (Figure 2c), including three material compositions (with Al_2_O_3_, with CuO, and without nanoparticles) and two temperature pressing levels.

### 2.2. Characterization of Resin

#### 2.2.1. Materials

The following materials related to resin characterization were used:

- Ford cup (Marte N4, São Paulo, Brazil);
- Urea-formaldehyde adhesive;
- Al_2_O_3_ nanoparticles (own laboratory production, Itapeva, Brazil);
- CuO nanoparticles (own laboratory production, Itapeva, Brazil);
- Meter device (Digimed DM22, São Paulo, Brazil).

#### 2.2.2. Methods

Viscosity testing was conducted using a Ford cup with a 5.20 mm orifice under ASTM D1200-2005 [23] (Standard Test Method for Viscosity by Ford Viscosity Cup).

The pH values of all resin samples were determined through direct measurements using a pH meter device (Digimed DM22).

To determine the gel time of each sample, 5 g of adhesive was utilized. Each adhesive was placed in a test tube and immersed in glycerin at 130 °C. A glass rod was continuously moved through the adhesive to induce gelation. The time taken for gelation in each sample was recorded as the gel time value.

### 2.3. Characterization of Physical and Mechanical Properties and Statistical Analysis

#### 2.3.1. Materials

The following materials related to physical–mechanical tests were used:

- Pachymeter (Mitutoyo Absolute 150 mm, Jundiaí, Brazil);
- Micrometer (Mitutoyo Digital 25 mm, Jundiaí, Brazil);
- Analytical digital scale (Marte 0.2 g precision, São Paulo, Brazil);
- Universal testing machine (EMIC 300T, Curitiba, Brazil);
- Muffle furnace (Marconi MA035, Piracicaba, Brazil).

#### 2.3.2. Methods

Regarding the physical and mechanical tests carried out to characterize these panels, the evaluation was based on density, moisture content, thickness swelling, static bending, and perpendicular tensile strength according to specifications given by the ABNT NBR 14810-3:2018 [22], including the dimensions and quantities of specimens to be characterized. Thus, 10 specimens were used for each test. In turn, a thermal conductivity test was carried out following the recommendations of the ASTM E1530:2011 [24]. For this, panel specimens 50 mm in diameter were used, with a total of three samples for each treatment.

After the characterization of particleboards, the results were statistically analyzed with an analysis of variance (ANOVA) at a 5% significance level to test for the existence of a significant difference between the means of the respective results.

Tukey’s test was then performed, also with 5% significance, to analyze the interactions. This analysis was carried out through R software version 3.2.0. In the result tables with statistics, the letter “A” denotes the group with the highest average value, “B” is the group with the second-highest average value, and so on.

## 3. Results and Discussion

### 3.1. Characterization of Nanoparticles

**Figure 3:**
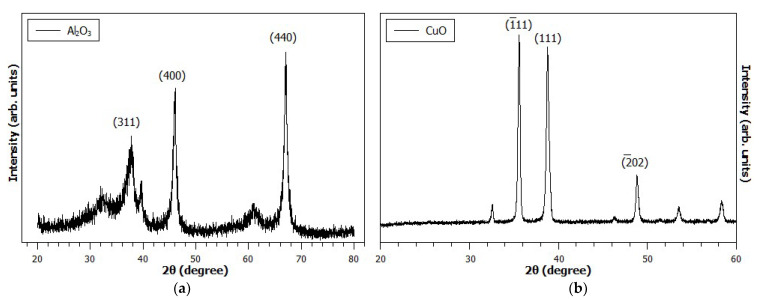
Diffractograms according to nanoparticles: (**a**) Al_2_O_3_ and (**b**) CuO.

The analysis of sizes and structures of the nanoparticles involved the characterization through X-ray diffraction. Figure 3 presents the diffractogram obtained from the analysis of Al_2_O_3_ and CuO nanoparticles. The diffractogram indicates the characteristic reflections of the γ-alumina phase, verifying the typical peaks of this cubic structure presented by the ICDD (International Center of Diffraction Data) with the reference number JCPDS 010-0425, which confirms the formation of crystalline γ-alumina.

The peaks have higher intensities. However, they are slightly broadened due to the nature of the crystallite size of these materials. Scherrer equation analysis of the average crystallite size for γ-alumina—from the full width at half maximum (FWHM) for the (311), (400), and (440) peaks found 7, 15, and 26 nm sizes, respectively—proves the synthesis of a nanocrystalline material. Likewise, by analyzing the ICSD database (Inorganic Crystal Structure Database), the presence of a single phase for copper II oxide with a monoclinic structure (ICSD reference number 16025) was verified. Thereby, Scherrer’s equation indicates a size of 37 nm, which also proves the synthesis of a nanocrystalline material.

### 3.2. Characterization of Resin

The pH, viscosity, and gel time properties of the resin are presented in Table 1.

From the results presented in Table 1, it is possible to state that the pH does not show a significant statistical difference with the addition of the nanomaterial. As verified in some previous studies, the resin presented a basic pH [6,18]. Gelatinization after 10 min was not found for any of the variations studied. This fact has already been observed by Silva et al. [25]. Regarding viscosity, the addition of nanoparticles increased the resin viscosity, which influenced the interaction between the matrix and reinforcement.

### 3.3. Characterization of Physical Properties

After the nanometric characterization of raw materials and physical tests of the panels, the results were compared with the standard values of ABNT NBR 14810:2018 (class P2) [26], EN 312:2003 [27], and ANSI 208-1:2016 (class M1) [28] for density, moisture, and thickness swelling. Non-structural panels measuring 13 to 20 mm thick were taken as a reference, for internal uses. All panels below this value were considered references and were classified as medium-density particles (MDPs). As shown in Table 2, the results obtained for panel density with respective coefficients of variation are presented with the ranges of values given by these standards valid in Brazil, Europe, and North America. Our results remained within the ranges from Brazilian and European standard documents, although they slightly exceeded the maximum average value stipulated for the North American market.

From Table 2, the density results also demonstrate some similarity to other studies, although they were lower than the average values reported by the literature. For example, Lima et al. [12], when manufacturing medium-density particleboards made with zinc oxide nanoparticles (ZnO), glued with urea-formaldehyde resin, and pressed at 180 °C in different proportions (0.5% and 1%), achieved average densities of 720.31 kg/m^3^ and 749.98 kg/m^3^, with no statistical difference between their results. It is worth highlighting that the experimental density of panels was lower than the nominal density (as indicated in the methodology), as manufacturing losses were verified in the particle gluing and mat formation stages.

Following the same presentation of our results and the ranges from standards as exemplified in Table 2, Table 3 analyzes the moisture content of particleboards pressed in two temperature levels and configured in three material compositions (with Al_2_O_3_, with CuO, and without nanoparticles).

Regarding the moisture analysis of the particleboards developed in this study, it was possible to verify that all treatments met the values referenced by the ABNT [26], with no statistical difference among the results from Table 3. All results were close to the minimum moisture content suggested by this same standard document.

These results were also similar to those reported by other scientific studies. Lima et al. [12] presented moisture contents of 6.18 and 6.24% for wood particle panels with 0.5% and 1% ZnO (urea-formaldehyde resin and 180 °C pressing temperature), while Silva et al. [8] obtained an average of 6.61% moisture under the same conditions. These authors did not find any statistical difference.

Similar to the organization of the previous tables, Table 4 discloses the obtained results for thickness swelling after 24 h. For thickness swelling after 24 h, none of the panels met the specifications from Brazilian and European standards. Particleboards with aluminum oxide nanoparticles at 150 °C reached the worst performance in this property, while panels without nanoparticles and at 180 °C showed the best results, statistically differing from the others (Table 4).

For medium-density particleboards manufactured with 4% SiO_2_ nanoparticles (urea-formaldehyde resin and 160 °C pressing temperature), Valle [10] reached an average value of 36.46% for this same property after 24 h. In nanocellulose-treated particleboards (urea-formaldehyde resin and 160 °C pressing temperature), Cardoso et al. [29] verified thickness swelling values of 30.35% and 53.68% for 2% and 3% nanocellulose, respectively. They still confirmed a negative influence with the addition of nanoparticles.

Taghiyari and Bibalan [20] also obtained an increase in the 24 h swelling of wood particle panels (urea-formaldehyde resin and 200 °C pressing temperature) when adding 150 mL/kg of copper nanoparticles, which increased this property from 19.52% to 22.68%.

**Figure 4:**
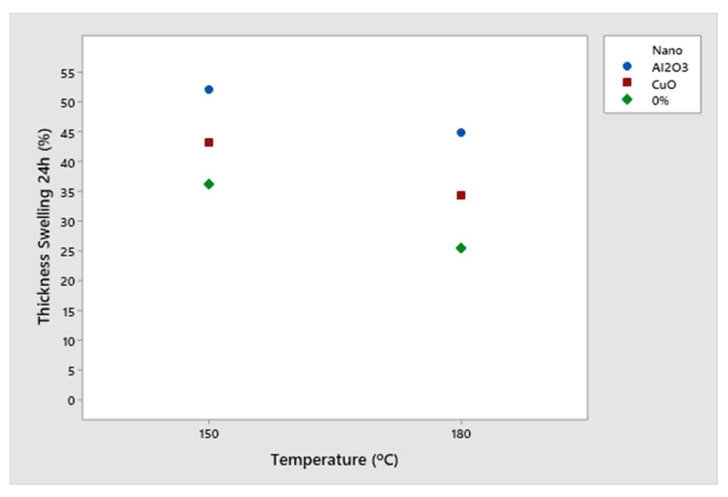
Interaction graph of results for thickness swelling after 24 h according to each panel composition.

Figure 4 illustrates the interactions between the treatments as well as possible trends presented by the addition of the nanoparticles.

Analyzing the interactions (Figure 4), it was possible to observe that—although the values for CuO were lower than for Al_2_O_3_—the addition of nanomaterials exhibited the same effect on the particleboards for this property, in which the pressing temperature was the greatest influential variable. Thus, a higher pressing temperature favors the curing of the polymeric adhesive, reducing thickness swelling values. It is also noteworthy that the percentage of nanoparticles used was smaller than in other research studies—for example, Zhang et al. [18] used up to 4% aluminum oxide nanoparticles.

Figure 4 illustrates the decrease in thickness swelling after water immersion for 24 h in all panels (without nanoparticles, with copper oxide, and with aluminum oxide). The greater the difference in values between the points for the same treatment, the greater the interaction between the factors. Occurred at a temperature of 180 °C, this reduction can be explained by the greater polymerization of the resin at higher temperatures, improving the performance of the panels in contact with water, that is, allowing better interaction of the resin with the wood particles, as confirmed by Lima et al. [12].

### 3.4. Characterization of Mechanical Properties

The same category of non-structural panel for internal uses, with a thickness between 13 and 20 mm, was considered for this analysis. The results were compared with the standard values of ABNT NBR 14810:2018 (class P2) [26], EN 312:2003 [27], and ANSI 208-1:2016 (class M1) [28] for commercial use in dry environments concerning the MOE and MOR.

Table 5 presents the results obtained for the modulus of elasticity (MOE), including respective coefficients of variation, and the ranges of values given by standard documents from Brazil, Europe, and North America. Only the particleboard pressed at 180 °C and produced with CuO nanoparticles did not reach the minimum expected for the European standards. However, it met the expected modulus of elasticity for the Brazilian and North American markets (Table 5). All configurations produced at a 150 °C pressing temperature exceeded the minimum standardized conditions for the modulus of elasticity.

Although almost all treatments were superior to the minimum modulus of elasticity specified by these standards (Table 5); only particleboards made with Al_2_O_3_ nanoparticles pressed at 180 °C differed statistically.

Obtained in static bending test, the modulus of rupture (MOR) is analyzed in Table 6. All panels pressed at 180 °C exceeded the minimum modulus of rupture given by the standards under consideration. In contrast, all panels pressed at 150 °C only met the minimum requirements for the North American region. All particleboards are close to the minimum performance expected by Brazilians, Europeans, and North Americans.

It is known that there is a relationship between mechanical resistance and density, and this is not influenced by the addition of nanoparticles. Thus, it was already expected that there would not be visible changes in the modulus of rupture values. The results showed a small difference between the treatments, while the reduced percentage of nanoparticles added to the adhesive can be considered as previously mentioned.

It is possible to highlight that the panels produced with copper oxide at 150 °C were statistically equivalent to the panels produced without nanoparticles at 180 °C, suggesting that the use of this nanomaterial allows a reduction in the pressing temperature. This gain is both economically and environmentally favorable in terms of reducing energy consumption in pressing, which is the most expensive stage in panel manufacturing. In future studies with higher percentages of nanoparticles, better results are expected.

**Figure 5:**
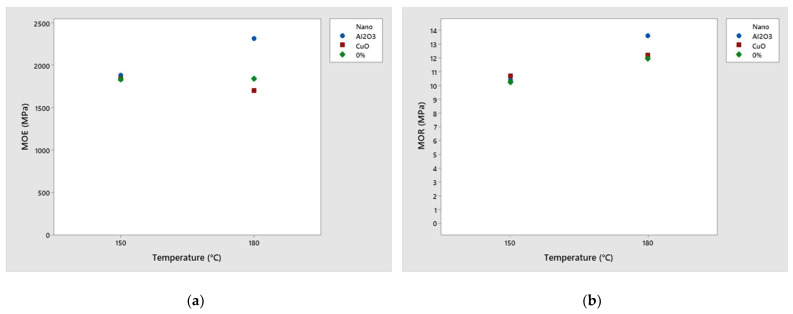
Interaction graph of results according to each panel composition for (**a**) modulus of elasticity and (**b**) modulus of rupture.

Through the interactions, it is possible to observe that the modulus of elasticity for CuO nanoparticles showed a decreasing trend with increasing temperature (Figure 5a).

Also, Taghiyari and Bibalan [20] obtained a reduction in the MOE in panels produced with urea-formaldehyde at 200 °C, as the addition of 150 mL/kg of copper nanoparticles decreased this property from 1830 MPa to 1775 MPa. In Figure 5, the interaction graph shows differences in behavioral trends with increasing temperature for the three treatments analyzed. For copper nanoparticles, there was a decrease, although it was not significant (Table 5). The better performance verified in the treatment with aluminum nanoparticles can be justified due to the increase in thermal conductivity with the addition of the nanomaterial, which was also observed by Gupta et al. [19]. The effect of temperature increasing on the MOE for the treatment without nanoparticles does not present a significant change—this is justified by the fact that UF resin can cure efficiently from 150 °C [5].

Regarding the modulus of rupture (Figure 5b), all treatments exhibited an upward trend with increasing temperature, with a more notable effect observed for Al_2_O_3_ nanoparticles. As observed for the MOE, the increase in thermal conductivity resulting from the use of Al_2_O_3_ nanoparticles promoted a significant increase in the MOR property. Other treatments demonstrated non-significant increases.

Comparing our results to other studies, Valle [10] obtained MOR values close to 12.25 MPa, finding no statistical difference, whereas Lima et al. [5] reached an average value of 13.3 MPa with ZnO added to the urea-formaldehyde adhesive. Neither study showed statistical differences. Also, Taghiyari and Bibalan [20] did not obtain an increase in the modulus of rupture for particulate panels produced with urea-formaldehyde and pressed at 200 °C when adding 100 mL/kg of copper nanoparticles, although the 150 mL/kg addition showed an increase from 11.56 MPa to 12.43 MPa in this same mechanical property.

### 3.5. Analysis of the Variation in Pressing Temperature

**Figure 6:**
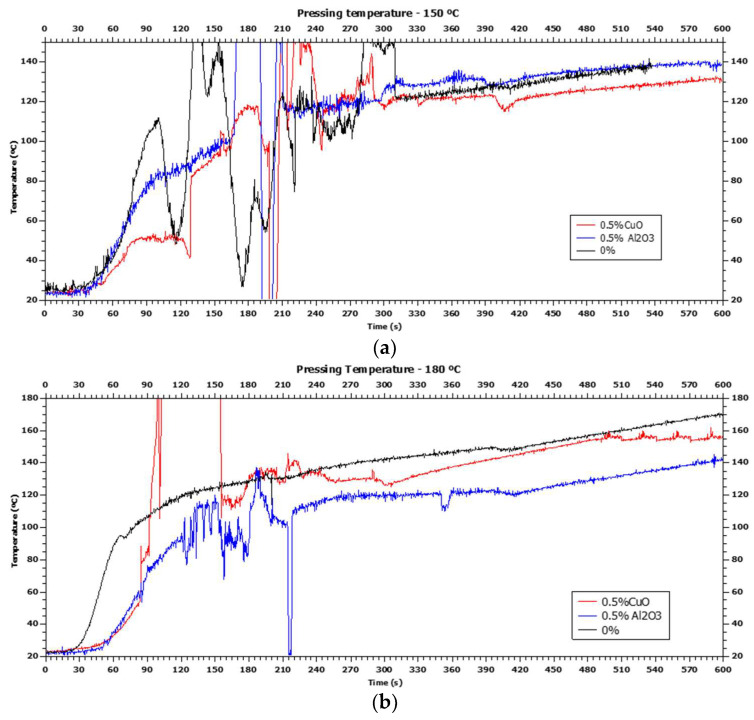
Graphs of variation in pressing temperature at (**a**) 150 °C and (**b**) 180 °C.

Sequentially to the nanometric evaluation of the material and physical–mechanical characterizations of the panels reported in the previous subitems, Figure 6a,b shows the graphs obtained from the average panel data after hot pressing at 150 °C and 180 °C.

It was possible to observe that both the particleboards produced without nanoparticles and the panels manufactured with the addition of nanoparticles (aluminum oxide and copper oxide) did not reach the expected pressing temperatures (150 °C in Figure 5a and 180 °C in Figure 5b), which could influence in the polymerization of the resin, as well as affect the adequate curing of the eucalypt wood panels developed in this study.

When adding copper oxide nanoparticles, the pressing temperature in the innermost layer of the panel was reduced in this exact location of thermocouple measuring, which did not occur when adding aluminum oxide nanoparticles (Figure 5a). In turn, both additions of nanoparticles influenced the protocols for manufacturing different panel compositions, as the presence of these nanoparticles reduced the pressing temperature in Figure 4.

Aluminum oxide has greater thermal insulation capacity as this treatment has lower thermal conductivity than copper oxide. According to Barea [30], when the temperature of aluminum oxide increases, its thermal conductivity decreases and, therefore, it may lead to a 5 °C difference between the final pressing temperatures for two analyses (Figure 4 and Figure 5).

At 150 °C, it is noted that particleboards produced with aluminum oxide reached the highest temperature. At 180 °C, the panel without nanoparticles reached the highest temperature, which approached the ideal temperature. In this case, this nanoparticle-free treatment also obtained the best thickness swelling properties. At 180 °C, it is also possible to observe that the panels produced with nanoparticles presented slower heating, which is justified by the refractory effect present in the oxides. The added nanomaterials may also have interfered with the physical and mechanical properties by retaining greater heat and, therefore, may have affected the polymerization of the urea-formaldehyde adhesive.

Regarding copper oxide nanoparticles, when comparing the two graphs in Figure 5, practically the same variation in final pressing temperature is observed in particleboards without nanoparticles (25 and 30 °C, respectively), presenting intermediate performances in their physical and mechanical properties compared to other treatments.

From effects of copper oxide nanoparticle addition, Taghiyari and Bibalan [20] concluded that, to obtain better heat transfer performance of copper nanoparticles in wood particleboards, it is desirable to increase their quantity. They noticed a reduction in the modulus of elasticity, justifying that it may have been caused by a negative influence on the bonding between the wood particles and the polymeric resin.

For higher levels of nanoparticles, better physical–mechanical properties and lower energy consumption were confirmed by [18]. Therefore, the observed effects can be attributed to the temperature gradient between both surfaces and the core in the particleboard during hot pressing, as explained by Zhang et al. [6], where the type and relative balance of covalent and ionic bonds in the polymeric resin structure may differ in these different regions of composite panels, that is, externally and internally.

### 3.6. Analysis of Thermal Conductivity

As shown in Table 7, thermal conductivity was analyzed. The added nanoparticles led to greater values for the lower temperature level at 150 °C, a condition opposite to the panel without nanoparticles. Particleboard containing copper oxide showed higher thermal conductivity than panels with aluminum oxide. 

Thermal conductivity ranged from 0.137 W/mK to 0.164 W/mK for our particleboards under evaluation (Table 7).

Our results also suggest that tested panels may serve as efficient thermal insulating materials, as they are in accordance with the observations of Bonduelle [31]. Panels with insulating performance are recommended for construction uses, where indoor spaces can be more pleasant than the external environment. Values between 0.09 W/mK and 0.197 W/mK were found by Çavuş et al. [32] in their study with different wood species, while Binici et al. [33] identified values between 0.075 W/mK and 0.1588 W/mK for corn-straw panels. This better thermal performance was possible due to the addition of nanoparticles. The added nanomaterials increased the panel conductivity, as it was 20% higher in panels produced at 150 °C (Table 7). Therefore, particleboards produced without nanoparticles require a higher pressing temperature to achieve the same effect provided by the nanomaterials, especially copper oxide. In this way, the nanoparticles allow the use of a lower pressing temperature for better thermal conduction.

## 4. Conclusions

As shown by our results obtained in physical tests to characterize the particleboards, the nanoparticles did not provide a significant improvement in thickness swelling properties, and they did not interfere with other properties. This can be justified by the 0.5% content of nanoparticles, which led to a limited influence on the material. For mechanical tests, the best performances are related to the increase in temperature, where a higher pressing temperature contributes to the heat transfer during pressing and assists the polymerization of the adhesive and curing of panels, which results in more efficient chemical bonds and the improvement of mechanical properties.

Specifically, we concluded the following:

- For both temperatures, our particleboards were suitable for internal uses, as they were in accordance with Brazilian and European standards for particleboards.
- An increase in temperature from 150 °C to 180 °C allowed better physical and mechanical results for all treatments, reaffirming that the innermost region of the panel reached a higher temperature for polymerization and curing.
- The thermal conductivity of panels with nanoparticles was higher than panels without them, although the increase in temperature reduced this effect. Oxide nanoparticles may have provided a refractory effect common to ceramic materials, which makes it preferable to opt for lower pressing temperatures, especially aluminum oxide, which is positive in terms of reducing energy consumption in a costly stage of panel production.
- Thermal conductivity and pressing temperature evaluations suggest that, in certain situations, the nanoparticles studied can contribute to better heat transfer to the innermost region of the panel. However, an impaired interaction between the wood particles and the polymeric formaldehyde-based adhesive was observed, provided by the addition of more environmentally friendly nanomaterials, which did not allow significant improvements in physical and mechanical properties. This effect can be justified by the increase in the viscosity of the resin when the nanomaterial is added, indicating a poor interaction of the aluminum oxide and copper oxide nanoparticles with the polymers.
- The percentage of 0.5% nanoparticles produced by the sol–gel protein methodology in use was lower than studies found in the literature, as these publications obtained better results in the physical–mechanical performance of panels with commercial nanoparticle contents ranging from 2 and 4%. The lower performance obtained can be justified by the higher viscosity of the adhesive when nanoparticles were added, which impaired the interaction between the particles and the polymeric adhesive.

In light of the above conclusions, different levels of nanoparticles and percentages of nanoparticles between external and internal layers are suggested for future studies, as these topics can be regarded to identify better heat transfers to allow a more significant influence of nanoparticles. In addition, new alternatives for the application of nanoparticles ought to be studied to obtain better results, including the insertion of other nanomaterials, different pressing temperature levels, structural adhesives, and other composite products such as plywood, fiberboards, structural particleboards, and glued engineered wood products.

## Figures and Tables

**Table 1:** pH, viscosity, and gel time properties.

Resin	Obtained Results	
	pH	Viscosity (Cp)
Urea-formaldehyde (UF)	8.51 ^A^	435.60 ^A^
UF + nano Al_2_O_3_	8.31 ^A^	572.19 ^B^
UF + nano CuO	8.64 ^A^	638.88 ^B^

^A^: group with the highest average value; ^B^: group with the second-highest average value.

**Table 2:** Density: results and comparison with values given by standards.

Temperature Pressing	150 °C	180 °C
Without nanoparticles	661.36 ^1,A^ (5.98 ^2^)	667.43 ^1,A^ (6.45 ^2^)
Al_2_O_3_ nanoparticle	674.72 ^1,A^ (6.03 ^2^)	707.57 ^1,A^ (5.28 ^2^)
CuO nanoparticle	682.64 ^1,A^ (6.59 ^2^)	665.68 ^1,A^ (7.48 ^2^)
Brazil ABNT [26]	551 to 750 ^1^	
Europe EN [27]	500 to 800 ^1^	
North America ANSI [28]	640 to 800 ^1^	

^1^ panel density (in kg/m^3^); ^2^ coefficient of variation; ^A^: group with the highest average value.

**Table 3:** Moisture content: results and comparison with values prescribed by standards.

Temperature Pressing	150 °C	180 °C
Without nanoparticles	6.49 ^1,A^ (4.93 ^2^)	6.41 ^1,A^(4.70 ^2^)
Al_2_O_3_ nanoparticle	6.65 ^1,A^ (2.11 ^2^)	6.51 ^1,A^ (5.53 ^2^)
CuO nanoparticle	6.56 ^1,A^ (2.90 ^2^)	6.15 ^1,A^ (5.85 ^2^)
Brazil ABNT [26]	5 to 13 ^1^	
Europe EN [27]	-	
North America ANSI [28]	-	

^1^ moisture content (in %); ^2^ coefficient of variation; ^A^: group with the highest average value.

**Table 4:** Thickness swelling: results and comparison with values prescribed by standards.

Temperature Pressing	150 °C	180 °C
Without nanoparticles	36.31 ^1,B^ (16.33 ^2^)	25.57 ^1,C^ (10.60 ^2^)
Al_2_O_3_ nanoparticle	52.18 ^1,A^ (9.37 ^2^)	44.95 ^1,A,B^ (1.91 ^2^)
CuO nanoparticle	43.21 ^1,A,B^ (13.75 ^2^)	34.33 ^1,B^ (11.97 ^2^)
Brazil ABNT [26]	<22 ^1^	
Europe EN [27]	<16 ^1^	
North America ANSI [28]	-	

^1^ thickness swelling after 24 h (in %); ^2^ coefficient of variation; ^A^: group with the highest average value; ^B^: group with the second-highest average value; ^C^: group with the third-highest average value.

**Table 5:** Modulus of elasticity: results and comparison with values given by standards.

Temperature Pressing	150 °C	180 °C
Without nanoparticles	1831 ^1,B^ (18.12 ^2^)	1840 ^1,B^ (13.22 ^2^)
Al_2_O_3_ nanoparticle	1880 ^1,B^ (12.01 ^2^)	2316 ^1,A^ (11.47 ^2^)
CuO nanoparticle	1845 ^1,B^ (8.35 ^2^)	1703 ^1,B^ (19.56 ^2^)
Brazil ABNT [26]	>1600 ^1^	
Europe EN [27]	>1800 ^1^	
North America ANSI [28]	>1550 ^1^	

^1^ modulus of elasticity (in MPa); ^2^ coefficient of variation; ^A^: group with the highest average value; ^B^: group with the second-highest average value.

**Table 6:** Modulus of rupture: results and comparison with values given by standards.

Temperature Pressing	150 °C	180 °C
Without nanoparticles	10.24 ^1,B^ (4.18 ^2^)	11.97 ^1,A,B^ (17.46 ^2^)
Al_2_O_3_ nanoparticle	10.42 ^1,B^ (15.54 ^2^)	13.64 ^1,A^ (14.74 ^2^)
CuO nanoparticle	10.70 ^1,A,B^ (16.92 ^2^)	12.24 ^1,A,B^ (14.71 ^2^)
Brazil ABNT [26]	>11.00 ^1^	
Europe EN [27]	>11.50 ^1^	
North America ANSI [28]	>10.00 ^1^	

^1^ modulus of rupture (in MPa); ^2^ coefficient of variation; ^A^: group with the highest average value; ^B^: group with the second-highest average value.

**Table 7:** Thermal conductivity: results.

Temperature Pressing	150 °C	180 °C
Without nanoparticle	0.137 ^1,B^ (11.67 ^2^)	0.153 ^1,A,B^ (3.27 ^2^)
Al_2_O_3_ nanoparticle	0.162 ^1,A^ (3.08 ^2^)	0.153 ^1,A,B^ (9.15 ^2^)
CuO nanoparticle	0.164 ^1,A^ (5.49 ^2^)	0.158 ^1,A,B^ (1.93 ^2^)

^1^ thermal conductivity (in W/mK); ^2^ coefficient of variation; ^A^: group with the highest average value; ^B^: group with the second-highest average value.

## Data Availability

Data are contained within the article.

## References

[1] UNECE/FAO (2023). COFFI Market Forecasts. https://unece.org/forests/coffi-market-forecasts.

[2] De Araujo V., Vasconcelos J., Lahr F., Christoforo A. (2022). Timber forest products: A way to intensify global bioeconomy from bio-materials. Acta Fac. Xylologiae Zvolen.

[3] Sakai S., Chen S., Matsuo-Ueda M., Umemura K. (2023). Curing behavior of sucrose with p-toluenesulfonic acid. Polymers.

[4] Iwakiri S. (2005). Painéis de Madeira Reconstituída [Panels of Reconstituted Wood].

[5] Lima F.O., Silva L.C.L., Campos C.I., Christoforo A.L., Lahr F.A.R. (2018). Pressing time influences on physical and mechanical properties of MDP panels. Sci. For..

[6] Zhang B., Chen X., Pizzi A., Petrissans M., Dumarcay S., Petrissans A., Zhou X., Du G., Colin B., Xi X. (2023). Highly branched tannin-tris(2-aminoethyl)amine-urea wood adhesives. Polymers.

[7] Galdino D.S., Kondo M.Y., De Araujo V.A., Ferrufino G.L.A.A., Faustino E., Santos H.F., Christoforo A.L., Luna C.M.R., Campos C.I. (2023). Thermal and gluing properties of phenol-based resin with lignin for potential application in structural composites. Polymers.

[8] Silva L.C.L., Lima F.O., Favarim H.R., Campos C.I. (2019). Heat transfer and physical-mechanical properties analysis of particleboard produced with ZnO nanoparticles addition. BioResources.

[9] Taghiyari H.R., Rangavar R., Bibalan O.F. (2011). Effect of nano-silver on reduction of hot-pressing time and improvement in physical and mechanical properties of particleboard. BioResources.

[10] Valle A.C.M. (2015). Análise das Propriedades Físicas e Mecânicas de Painéis MDP de Madeira de Eucalipto Com Adição de Nanopartículas de Sílica ao Adesivo Uréia-Formaldeído [Analysis of the Physical and Mechanical Properties of MDP of Eucalypt Wood with Addition of Silica Nanoparticles to Urea-Formaldehyde Adhesive].

[11] da Silva A.P.S., Ferreira B.S., Favarim H.R., Silva M.F.F., Silva J.V.F., Azambuja M.A., Campos C.I. (2019). Physical properties of medium density fiberboard produced with the addition of ZnO nanoparticles. BioResources.

[12] Lima F.O., Silva L.C.L., Ferreira B.S., Morais C.A.G., Bertolini M.S., Barreiros R.M., Azambuja M.A., Caraschi J.C., Favarim H.R., Campos C.I. (2022). Influence of the addition of Al_2_O_3_ nanoparticles and the duration of pressing on the physical properties of OSB panels. BioResources.

[13] Mallakpour S., Khadem E. (2015). Recent development in the synthesis of polymer nanocomposites based on nano-alumina. Prog. Polym. Sci..

[14] Yu J., Huang X., Wang L., Peng P., Wu C., Wu X., Jiang P. (2011). Preparation of hyperbranched aromatic polyamide grafted nanoparticles for thermal properties reinforcement of epoxy composites. RSC Polym. Chem..

[15] Cademartori P.H.G., Artner M.A., Freitas R.A., Magalhães W.L.E.M. (2019). Alumina nanoparticles as formaldehyde scavenger for urea-formaldehyde resin: Rheological and in-situ cure performance. Compos. Part B Eng..

[16] Roumeli E., Papadopoulou E., Pavlidou E., Vourlias G., Bikiaris D., Paraskevopoulos K.M., Chrissafis K. (2012). Synthesis, characterization and thermal analysis of urea–formaldehyde/nanoSiO_2_ resins. Thermochim. Acta.

[17] Tian H., Zhang Q., Pizzi A., Lei H., Wang J., Xi X. (2023). Adhesion properties and formaldehyde emissions of MnO_2_/UF nanocomposite adhesives. Int. J. Adhes. Adhes..

[18] Zhang R., Jin X., Wen X., Chen Q., Qin D. (2018). Alumina nanoparticle modified phenol-formaldehyde resin as a wood adhesive. Int. J. Adhes. Adhes..

[19] Gupta A., Kumar A., Sharma K.V., Gupta R. (2018). Application of high conductive nanoparticles to enhance the thermal and mechanical properties of wood composite. Mater. Today Proc..

[20] Taghiyari H.R., Bibalan O.F. (2013). Effect of copper nanoparticles on permeability, physical and mechanical properties of particleboard. Eur. J. Wood Wood Prod..

[21] Lima Z.M.F. (2011). Nova Rota de Síntese de Nanopartículas de NiMn_2_O_4_ Usando o Método Sol-Gel Protéico [New Route of Synthesis Nanoparticles NiMn_2_O_4_ Method Using Sol-Gel Protein]. Master’s Thesis.

[22] (2013). Chapas de Madeira Aglomerada—Parte 3: Métodos de Ensaio [Wood Particleboard—Part 3: Test Methods].

[23] (2023). Standard Test Method for Viscosity by Ford Viscosity Cup.

[24] (2011). Standard Test Method for Evaluating the Resistance to Thermal Transmission of Materials by the Guarded Heat Flow Meter Technique.

[25] Silva G.C., Lelis R.C.C., de Oliveira G.L., da Silva B.C., da Lossano W.C.S., dos Abreu H.S. (2019). Propriedades de adesivo aplicado em painéis a partir da substituição por lignossulfonato do processo sulfito. Ciênc. Florest..

[26] (2018). Chapas de Madeira Aglomerada—Parte 2: Requisitos [Wood Particleboard—Part 2: Requirements].

[27] (2003). Particleboards. Specifications.

[28] (2016). Particleboard.

[29] Cardoso G.V., Teixeira F.P., Ferreira E.S. Nanocelulose como catalisador de ureia-formaldeído para produção de painéis aglomerados [Nanocellulose as a urea-formaldehyde catalyst for particleboard production]. Proceedings of the 15th Brazilian Conference of Woods and Timber Structures (XV Ebramem).

[30] Barea R., Belmonte M., Osendi M.I., Miranzo P. (2003). Thermal conductivity of Al_2_O_3_/SiC platelet composites. J. Eur. Ceram. Soc..

[31] Bonduelle G.M. (2017). Propriedades Térmicas da Madeira [Thermal Properties of Wood].

[32] Çavuş V., Şahin S., Esteves B., Ayata Ü. (2019). Determination of thermal conductivity properties in some wood species obtained from Turkey. BioResources.

[33] Binici H., Aksogan O., Demirhan C. (2016). Mechanical, thermal and acoustical characterizations of an insulation composite made of bio-based materials. Sustain. Cities Soc..

